# Reversible dementia due to neurocysticercosis: Improvement of the racemose type with antihistamines

**DOI:** 10.1590/S1980-57642015DN91000014

**Published:** 2015

**Authors:** Gislaine Cristina Lopes Machado-Porto, Leandro Tavares Lucato, Fábio Henrique de Gobbi Porto, Evandro Cesar de Souza, Ricardo Nitrini

**Affiliations:** 1 MD, PhD student, Instituto de Radiologia do Hospital das Clínicas da Faculdade de Medicina da Universidade de São Paulo, São Paulo SP, Brazil. Department of Radiology, A.C.Camargo Cancer Center, São Paulo SP, Brazil; 2 MD, PhD, Neuroradiologist, Instituto de Radiologia do Hospital das Clínicas da Faculdade de Medicina da Universidade de São Paulo, São Paulo SP, Brazil. Centro de Diagnóstico Brasil, São Paulo SP, Brazil; 3 MD, PhD student, Behavioral and Cognitive Neurology Unit, Department of Neurology Cognitive Disorders Reference Center (CEREDIC), HC/FMUSP, São Paulo, Brazil; 4 MD, PhD, Medical Assistant, Head of Neurological Radiosurgery Group, HC/FMUSP, São Paulo SP, Brazil; 5 MD, PhD, Full Professor, Behavioral and Cognitive Neurology Unit, Department of Neurology Cognitive Disorders Reference Center (CEREDIC), HC/FMUSP, São Paulo SP, Brazil.

**Keywords:** neurocysticercosis, racemose form, Taenia solium, dexchlorpheniramine, dementia, reversible dementia, antihistamine

## Abstract

Infection of the human central nervous system (CNS) by the larvae of *Taenia solium*, termed neurocysticercosis (NCC), is endemic in most developing countries, where it is a major cause of acquired seizures and other neurological morbidity, including neuropsychiatric symptoms. However, despite its frequent manifestation, some findings, such as cognitive impairment and dementia, remain poorly understood. Less commonly, NCC may affect the ventricular system and subarachnoid spaces and this form is known as extraparenchymal neurocysticercosis. A particular presentation of the subarachnoid form is called racemose cysticercosis, which has a progressive pattern, frequently leads to hydrocephalus and can be life-threatening. Here we review a case of the racemose variety of cysticercosis, complicated by hydrocephalus and reversible dementia, with remission of symptoms after derivation and that remained stable with use of dexchlorpheniramine. We discuss the challenges in diagnosis, imaging findings, treatment and follow-up of this disease.

## Abstract (pt)

A infecção do sistema nervoso central (SNC) pela larva da *Taenia solium*, intitulada neurocisticercose (NCC) é endêmica na maior parte dos países "em desenvolvimento", onde é a principal causa de convulsão adquirida, além de outras morbidades neurológicas, entre elas, sintomas neuropsiquiátricos. No entanto, apesar de manifestações neuropsiquiátricas serem frequentes, alguns achados, tais como comprometimento cognitivo e demência, continuam a ser mal compreendidos. Menos frequentemente, NCC pode afetar o sistema ventricular e espaços subaracnóideos e esta forma é conhecida como NCC extraparenquimatosa. Uma apresentação particular subaracnóidea, chamada cisticercose racemosa, é encontrada mais raramente, evolui de forma progressiva, associada a hidrocefalia e pode levar a morte. Neste artigo revisamos um caso da variedade racemosa de NCC, complicada com hidrocefalia e demência reversível que evoluiu com remissão dos sintomas após derivação ventricular e permaneceu estável com uso de dexclorfeniramina. Discutimos os desafios no diagnóstico, achados de imagem, tratamento e acompanhamento desta forma de doença.

## INTRODUCTION

Neurocysticercosis (NCC) denotes the infection of the human central nervous system (CNS) by the larvae form of the pork tapeworm *Taenia solium*. NCC is the most common parasitic infection of the central nervous system and a major cause of adult acquired epilepsy and other neurological morbidity in the developing world.^1,2^

Clinical manifestations correlate strongly with the stage of cysticercus biological evolution and occur mainly in the evolutive stage of impairment of parasite vitality that causes disruption of host-parasite equilibrium.^1,3,4^ Parenchymal disease is mostly benign, often characterized by seizures, while extraparenchymal disease is associated with hydrocephalus, and has a poorer prognosis.^2,5^ Racemose NCC, a rare variety, has variable presentation depending on its location, resulting in basal arachnoiditis, meningeal fibrosis, adhesions and impairment of CSF flow.^6^ NCC can manifest with neuropsychiatric symptoms, however, some clinical findings, such as cognitive impairment and dementia, remain poorly characterized, due to lack of controlled studies.^7,8^

The management of NCC should be individualized in relation to the type of NCC. Treatment approach depends on the main clinical presentation, the number, location, size and stage of parasites, as well as on the immune response of the host.^1,4,5^ Computed tomography (CT) and particularly magnetic resonance imaging (MRI), play a role providing useful information on the number and topography of lesions, their stage of involution, and the degree of inflammatory reaction of the host against the parasites. Also, it is known that the identification of vesicles with scolex in the brain is pathognomonic of NC, confirming definite diagnosis.^9-11^

Current treatment options involve symptomatic agents, anti-parasitic agents or surgery. Most authors agree that initial measures should focus on symptomatic management with later consideration of anti-parasitic therapy when appropriate.^4,5,12^

## CASE REPORT

A 63-year-old woman, housewife, with 4 years of education, was evaluated at the outpatient neurological unit due to gait disorder, cognitive decline and urinary incontinence in September 2008. Her history had begun about 19 years earlier, when she developed headache and fever. At that time, cerebrospinal fluid (CSF) revealed neurocysticercosis and she was treated with improvement of symptoms. Full information on the medications she received at that time was unavailable, but she received an anti-parasitic drug and corticosteroids. Three years later, she had another episode of severe headache with nausea and vomits, which was successfully treated with steroids, but since then she had had repeated episodes of headache and vomits every two to three years. She had to be hospitalized several times and since 2003 had been taking steroids orally (Deflazacort 6 mg to 30 mg) almost continuously. She also reported improvement of the headaches when submitted to lumbar punctures for CSF analysis.

In 2005, she started to show progressive gait disturbance and complain of forgetfulness. Her husband confirmed the memory decline. These disorders worsened and about 2 years ago she started to have urinary incontinence. Due to gait disturbances and memory decline she was unable to perform her usual house chores.

She had been operated for benign thyroid cysts in 2003 and treated with thyroxine since then.

At first interview, neurologic examination revealed an unsteady gait with widened base. She was unable to walk in tandem. There was lack of body balance with slight tendency for retropulsion. Fundoscopy revealed bilateral papilledema in regression. She scored 24 on the Mini-mental State Examination (MMSE). Her scores on the verbal fluency tests were very low (7 animals in one minute and 7 words starting with the letter "p" in one minute). The remainder of the neurologic examination was unremarkable.

**Figure 1:**
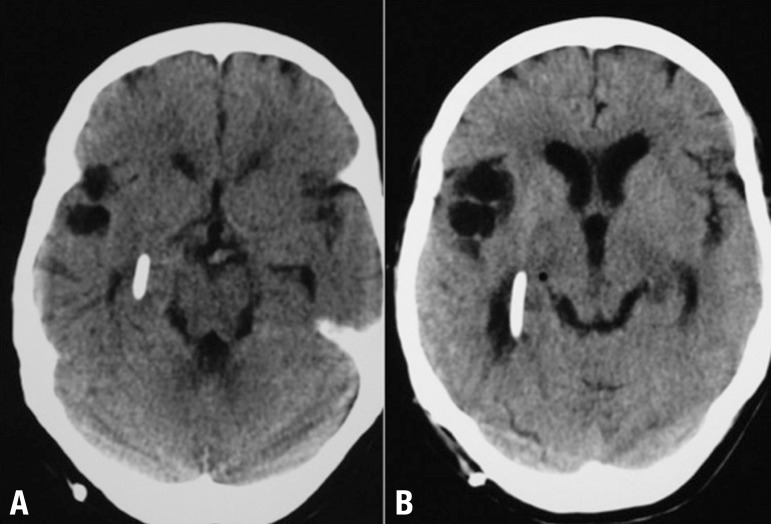
[A] Noncontrast brain CT-scan after first shunt derivation shows catheter tip of ventricular peritoneal shunt into right lateral ventricle, absence of ventricular dilatation and slight increase in right Sylvian fissure. [B] Follow-up noncontrast Brain CT-scan four years later after shunt replacement shows moderate dilatation of lateral ventricles and multiple cystic lesions in right Sylvian fissure.

A CSF analysis done in July 2008 disclosed 35 leukocytes (predominantly lymphomononuclear cells with 5% eosinophils), 77 mg/dL of proteins, with high concentration of gammaglobulin (27%; normal 7-14%), normal glucose and a positive test for neurocysticercosis on antibody-enzyme linked immunosorbent assay (Ag-ELISA). Serological tests for toxoplasmosis, cytomegalovirus and Epstein-Barr virus (both IgG and IgM), and HIV were all negative. In October 2008, MRI of the brain (not shown) had disclosed mild communicating hydrocephalus. At this time she was submitted to a ventricular shunt with a ventriculoperitoneal catheter and an almost immediate improvement was observed by the neurosurgeon (ECS). Also, a CT scan performed after the derivation showed resolution in hydrocephalus, and a slight increase in the right Sylvian fissure was depicted (Figure 1 A).

**Figure 2:**
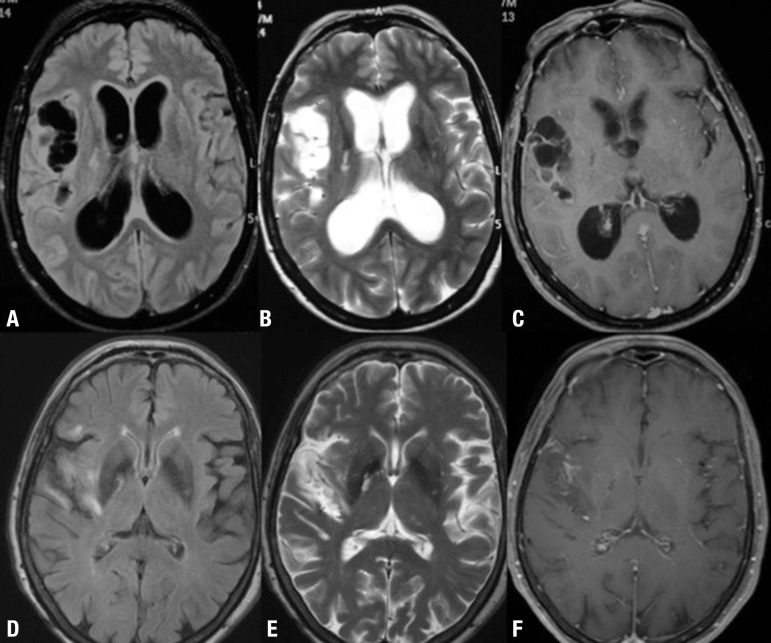
[A] Axial FLAIR, [B] Axial T2WI, [C] Axial T1W1 with gadolinium. [A, B] shows multiple cystic lesions without scolex in right Sylvian fissure. [C] gadolinium-enhanced MR image shows diffuse arachnoid enhancement surrounding the cysts. [D] Axial FLAIR, [E] Axial T2WI, [F] Axial T1W1 with gadolinium. [D, E and F] follow-up Brain MRI after second shunt replacement shows absence of hydrocephalus and slight enlargement of right Sylvian fissure. [F] slight enhancement in right Sylvian fissure, previously containing multiple cysts.

The patient did not return for consultation until May 2010 when she informed that after the ventricular shunt she had a rapid improvement of her urinary incontinence, gait disorder and memory problems and had resumed her normal activities. However, episodes of headache had reappeared together with somnolence, mental confusion and urinary incontinency about 8 months earlier. At examination, no papilledema was evident but a broad-based gait was observed. She scored 23 on the MMSE and was able to say only 6 animals in one minute. CT scan revealed increased ventricular enlargement (dilatation) (Figure 1 B). Brain MRI disclosed moderate hydrocephalus and the catheter tip of the ventricular peritoneal shunt into the right lateral ventricle. There were multiple cysts in the right Sylvian fissure, without scolex, and peripheral enhancement, whose appearance resembled a "bunch of grapes" (Figure 2, A, B and C).

Dexchlorpheniramine 6 mg daily (2 mg t.i.d) and dexamethasone were introduced. Dexamethasone was prescribed, in a tapering dose schedule, starting with 12 mg daily and ending after twenty days.

There was a slight improvement but in September 2010 she was operated to insert a new ventriculoperitoneal shunt. The patient experienced clinical improvement, with improvement of gait disorders, urinary incontinence and remission of papilledema. After three years of observation, the patient remains stable, in use of dexchlorpheniramine at 4 mg daily. Follow-up brain MRI was performed and showed resolution of hydrocephalus, remarkable reduction in the number of cysts in the Sylvian fissure, with persistent slight peripheral enhancement after gadolinium and high signal intensity on T2 / Flair (Figure 2, D, E and F).

In the last examination in March 2012, she had resumed her normal activities, without gait, cognitive or urinary problems and scored 30 on the MMSE.

## DISCUSSION

This report describes a case of the racemose form of neurocysticercosis, which had a complex clinical course, presenting with reversible dementia. The patient presented with progressive symptoms, clinical remissions and exacerbations, posing a treatment challenge. The patient was treated with anti-parasitic drugs, corticosteroids and ventricular shunts, improving after a new ventricular derivation and the use of dexchlorpheniramine. The underlying cause of reversible dementia in this case seems to have been hydrocephalus caused by racemose NCC.

Clinical presentation depends on number, location, size and stage of the parasites, as well as on the immune response of the host.^1^ The most common presentation of NCC is parenchymal, and it is usually associated with good prognosis. Most patients remain asymptomatic, although some develop seizures. Sometimes the cysts can grow and produce a mass effect.^1,4,11^

Cognitive disturbances are one of the most frequent manifestations of the disease, and some studies reported these symptoms in about 66 to 87% of patients with NCC.^7,8^ One such study found dementia in around 12.5% of patients with NCC and cognitive decline in approximately 45%.^7^ The most frequently impaired cognitive domains were executive functions, verbal memory and language and visuospatial skills. Cognitive impairment can be the result of multiple factors acting alone or in combination with mechanisms related to local inflammatory (parasitic) reaction, vascular lesion and immune-mediated reactions, or secondary epilepsy caused by NCC, as well antiepileptic drugs.^13^ This combined effect can disrupt frontal-parietal-temporal networks related to intellectual functioning in patients with vulnerable brains (because of repeated epileptic seizures, low educational levels, advanced age).^14^ Apparently, no correlation between cognitive disorders and number of NCC lesions on MRI has been established, however active disease and intracranial hypertension were associated with higher morbidity.^8,15^ Some reports have shown favorable outcomes in patients with cognitive impairment and NCC after appropriate therapy.^16^

Extraparenchymal NCC may be ventricular and/or subarachnoid. The relationship between hydrocephalus and NCC has long been reported.^17^ Extraparenchymal NCC may cause hydrocephalus by mechanical obstruction of the ventricles or the basal cisterns, either by the cysts themselves or by an inflammatory reaction (ependymitis, arachnoiditis).^6,18^ Ventricular NCC commonly affects the IV ventricle followed by III ventricle and lateral ventricles.^19,20^

Intraventricular NCC is often difficult to diagnose on CT. MRI, owing to its multiplanar capability and excellent depiction of tissue contrast, is more accurate than CT for assessing the degree of infection, location, and stage of the parasite in NCC, and especially intraventricular lesions. Furthermore, the detection of a scolex by CT or MRI within a cystic lesion, which is usually seen in the parenchymal form, is pathognomonic of NCC.^21,22^

Racemose NCC, a rare variety, is characterized by abnormal growth of cystic membranes, multiloculated, resembling a "bunch of grapes", with degeneration of the parasite's scolex, which occurs in the ventricles, Sylvian fissure and basal cisterns. It is associated with an intense inflammatory reaction, fibrosis and progressive thickening of the leptomeninges at the base of the brain. In this case, the patient had multiple cysts in the right Sylvian fissure, whose appearance resembled a "brunch of grapes" (Figure 2), consistent with racemose variety. Additionally, there was a new moderate hydrocephalus, and a second surgical approach for ventricular shunt was necessary, likely due to the presence of the membranes or inflammatory cells and proteins blocking the shunt (Figure 2). However other factors such as cysticercus in the Sylvian fissure preventing CSF circulation, recurrent meningitis with arachnoiditis and ependymitis could also have been responsible for the hydrocephalus. The underlying cause of reversible dementia in this case seems to have been hydrocephalus secondary to racemose NCC.

Analysis of CSF samples is an important parameter for the assessment and follow-up of patients with suspected NCC. There is a positive correlation between circulating parasite antigen and hydrocephalus secondary to neurocysticercosis, indicating the presence of live parasites or parasitic membranes in the ventricular cavities or basal cisterns.^2^ Some authors have demonstrated a significant relationship between the total number of lesions detected by MRI and the concentration of Taenia antigen (TA) detected using the ELISA technique.^3^ In this case, CSF analysis was positive for neurocysticercosis on ag-ELISA, probably due to parasitic membranes in the basal cisterns.

Treatment decisions in NCC should be individualized in relation to the type of NCC. Initial measures should focus on symptomatic management with later consideration of anti-parasitic therapy when appropriate.^5^

The introduction of praziquantel and albendazole as specific anti-parasitic agents was enthusiastically adopted by many segments of the medical community. However, many authors have questioned the value of these agents, and an intense controversy still exists.^1,4,24,25^ It is accepted that these specific anti-parasitic agents have even less efficacy in the treatment of the racemose variety of NCC.^26^ There are several hypotheses for this failure, largely hinging on host and parasite factors. It is believed that the cysticidal drugs may act differently depending on the stage of development of the parasite. In addition, these drugs may have less penetration into the subarachnoid space compared to brain parenchyma. Also, variability in plasma and CSF drug levels exists among patients due to individual differences in bioavailability.^26^

Furthermore, the low bio-availability of cysticidal drugs in the CSF may not only fail to inhibit parasitic growth, but may be conducive to antigen release that stimulates the immune response, leading to chronic inflammation.^26^ Therefore, in this case, cysticidal therapy was not performed after the appearance of multiple cysts in the sylvian fissure due to concerns over provoking acute brain inflammation and ependymitis from the death of cysts.

The use of steroids during anti-parasitic treatment is poorly studied. Published treatment regimens vary enormously in terms of the drug of choice, doses, and length of treatment. Most authors use a moderate amount of dexamethasone (usually 0.1 mg/k/d) from the day before starting on anti-parasitic treatment until the end of anti-parasitic treatment.^1,5^ However, some studies failed to show an effect in reducing the rate of seizure recurrence or lesion persistence on imaging studies in combined corticosteroids and anti-parasitic treatment.^5,27^ Also, some authors hold that the high levels of corticosteroids used to prevent complications due to severe CSF inflammation may also turn-off key immunological components crucial for parasite destruction.^20^ In this case the patient was initially treated with a course of anti-parasitic drugs, accompanied by the use of corticosteroids. Nevertheless, she had two relapses with hydrocephalus, despite the ventricular peritoneal shunt placement and the use of corticosteroids, demonstrating the progressive nature of the disease and difficulty managing the disease with available treatments.

The effectiveness of using dexchlorpheniramine (Polaramine) in NCC remains unclear and has given rise to considerable controversy. Dexchlorpheniramine is an antihistamine with anticholinergic properties, able to cross the blood-brain barrier and bind to the central H^1^ receptors.^28^ In some treatment regimens, antihistamines have been used as an adjuvant in anti-parasitic schemes to raise serum levels of these drugs.^24,25,29^ Moreover, some authors have used antihistamines as a substitute for steroids, since NCC is accompanied by prolonged, sometimes recurrent, periods of inflammatory status leading to long-term treatments, and consequently accumulation of steroidal side-effects.^24,25,30^ This replacement was based on knowledge about the immunoallergic mechanisms of the manifestations of NCC and on the fact that steroids do not inhibit the antigen-antibody reaction and the consequent release of histamine and other active substances, whereas antihistamines compete with histamine in their receptors and therefore impair its action. Despite the lack of controlled studies, these reports have described positive results in this treatment plan.^24,25,30^ After the second ventricular derivation, this patient remained stable with no further recurrent meningitis or hydrocephalus episodes with the continuous use of antihistamines. We hypothesize that the penetration of dexchlorpheniramine through the blood-brain barrier and its anti-inflammatory properties may play a role in the treatment of NCC, especially in the racemose presentation.

Surgical treatment for NCC is reserved for selected patients. It can be considered for intraventricular cysts, hydrocephalus due to racemose cysts or ependymitis, spinal cysticercosis, but is typically reserved for those with hydrocephalus. In some cases a surgical approach is used for the removal of ventricular cysts, especially when there is hydrocephalus due to a single cyst in the fourth ventricle.^5^ In racemose NCC, surgical treatment is usually limited to ventricular shunt and ventriculostomy. Recently, minimally invasive surgery (neuroendoscopy) has been added to open craniotomies to resect cysts from the ventricles, Sylvian fissure and other locations. Neuroendoscopy has been suggested as treatment, when available, for cyst extraction and fenestration of the III ventricle during the same procedure. However, the indications remain unclear and there is no controlled data supporting this treatment.^5,31^

This case illustrates an unusual presentation of reversible dementia caused by hydrocephalus secondary to NCC in its racemose form. Although cognitive impairment and neuropsychiatric disorders are frequent signs of NCC, they remain poorly understood, whereas in this case the cause of reversible dementia was clearly hydrocephalus. In addition, this case informs the discussion on the role of antihistamine in NCC, especially in its racemose form, which is often chronic, characterized by relapses and sometimes by prolonged use of steroids and even surgery. Although a complete understanding of the role of dexchlorpheniramine in the treatment of NCC cannot be gleaned through a single case, this report helps elucidate its possible mechanism of action.
